# Multiple machine learning methods aided virtual screening of Na_V_1.5 inhibitors

**DOI:** 10.1111/jcmm.17652

**Published:** 2022-12-27

**Authors:** Weikaixin Kong, Weiran Huang, Chao Peng, Bowen Zhang, Guifang Duan, Weining Ma, Zhuo Huang

**Affiliations:** ^1^ Department of Molecular and Cellular Pharmacology, School of Pharmaceutical Sciences Peking University Health Science Center Beijing China; ^2^ Institute for Molecular Medicine Finland (FIMM) HiLIFE, University of Helsinki Helsinki Finland; ^3^ Institute Sanqu Technology (Hangzhou) Co., Ltd. Hangzhou China; ^4^ ComMedX (Computational Medicine Beijing Co., Ltd.) Beijing China; ^5^ Department of Neurology Shengjing Hospital affiliated to China Medical University Shenyang China; ^6^ State Key Laboratory of Natural and Biomimetic Drugs, Department of Molecular and Cellular Pharmacology, School of Pharmaceutical Sciences Peking University Health Science Center Beijing China

**Keywords:** chemical inhibitors, machine learning, Na_v_1.5, privileged substructures

## Abstract

Na_v_1.5 sodium channels contribute to the generation of the rapid upstroke of the myocardial action potential and thereby play a central role in the excitability of myocardial cells. At present, the patch clamp method is the gold standard for ion channel inhibitor screening. However, this method has disadvantages such as high technical difficulty, high cost and low speed. In this study, novel machine learning models to screen chemical blockers were developed to overcome the above shortage. The data from the ChEMBL Database were employed to establish the machine learning models. Firstly, six molecular fingerprints together with five machine learning algorithms were used to develop 30 classification models to predict effective inhibitors. A validation and a test set were used to evaluate the performance of the models. Subsequently, the privileged substructures tightly associated with the inhibition of the Na_v_1.5 ion channel were extracted using the bioalerts Python package. In the validation set, the RF‐Graph model performed best. Similarly, RF‐Graph produced the best result in the test set in which the Prediction Accuracy (Q) was 0.9309 and Matthew's correlation coefficient was 0.8627, further indicating the model had high classification ability. The results of the privileged substructures indicated Sulfa structures and fragments with large Steric hindrance tend to block Na_v_1.5. In the unsupervised learning task of identifying sulfa drugs, MACCS and Graph fingerprints had good results. In summary, effective machine learning models have been constructed which help to screen potential inhibitors of the Na_v_1.5 ion channel and key privileged substructures with high affinity were also extracted.

## 1 INTRODUCTION

Voltage‐gated sodium channel subtype 1.5 (Na_V_1.5) is the major cardiac voltage‐gated sodium ion channel, which plays a vital role in the generation of the cardiac action potential and in the propagation of the electrical impulses in the heart.^1^ The role of Na_V_1.5 in the aetiology of numerous cardiac anomalies strongly suggests the proper regulation of the channel is critical for normal heart function. Also, the pivotal function of Na_V_1.5 in normal heart operation has been discovered by researching the genetic mutation of SCN5A located on chromosome 3p21 which encodes Na_V_1.5. Tan et al.^2^ found Na_V_1.5 is linked to congenital and drug‐acquired Long QT Syndrome (LQTS), Brugada Syndrome (BS), conduction disorders and sudden infant death syndrome. Other research has also indicated that this gene is also associated with disorder‐ventricular arrhythmia and dilatative cardiomyopathy.^3^

Cardiac toxicity of drugs has always been forefront for drug administration, and many non‐cardiac drugs, especially psychotropic drugs, can introduce ventricular fibrillation and syncope and sudden cardiac arrest (SCA) by reducing cardiac excitability through Na_V_1.5.^4^

At present, the patch‐clamp electrophysiological method is still the gold standard for ion channel drug screening. However, this method has disadvantages such as high technical difficulty, high cost and low speed. In this case, drug virtual screening based on computational methods can help to find drugs with higher specificity and bioactivity along with a higher speed and less consumption. Machine learning (ML) has become very popular recently, due to increased data availability and algorithmic methods, and has been employed in drug design and screening. ML approaches provide a set of tools that can use abundant, high‐quality data to solve discovery and decision‐making for well‐specified questions. ML models are based on existing data to do predictions which can accelerate the new drug discovery process, which have been applied in all stages of drug discovery.^5^, ^6^, ^7^, ^8^ Examples include the identification of prognostic biomarkers,^9^, ^10^ drug repurposing,^11^, ^12^, ^13^, ^14^ and analysis of side effect.^15^ With the development of high‐throughput screening technology, countless meaningful experimental data are being produced to the benefit of future work using computer‐dependent drug design.

In this study, building ML classification models are established based on molecular features to predict chemicals that have a high affinity of Na_V_1.5. A comparison with the graph convolutional neural network method is also made to find the most effective prediction method.

## 2 MATERIALS AND METHODS

### 2.1 Data preparation

In this study, the inhibition data were acquired from the ChEMBL Database (ChEMBL ID:CHEMBL1980). The download date was 3 October 2020. The Homo sapiens Na_V_1.5 ion channel was used as the target and IC_50_ as the experimental method. The data preparation process was shown in Figure S1. After deleting duplicate molecules and only keeping experimentally verified molecules, there were 1957 diverse compounds left which were encoded into a standard simplified molecular‐input line‐entry system (SMILES).

Then, according to other researchers' previous work,^16^, ^17^, ^18^ 30,000 nM was used as the threshold to divide molecules into positive molecules and negative molecules. The molecules with IC50 values less than 30,000 nM were tagged to the label “1” which represented the molecules that were able to inhibit Na_V_1.5. In contrast, the negative molecules were tagged to the label “0.” In this step, we obtained 1785 positive molecules and 172 negative molecules. To exclude the polymers and make sure selected molecules were drug‐like, the compounds with atom numbers over 120 were deleted, and then only molecules confirmed by Linpinski's rule of 5 were left. After the above process, the positive molecules were reduced to 1558 molecules and the negative molecules were reduced to 96 molecules.

**FIGURE 1:**
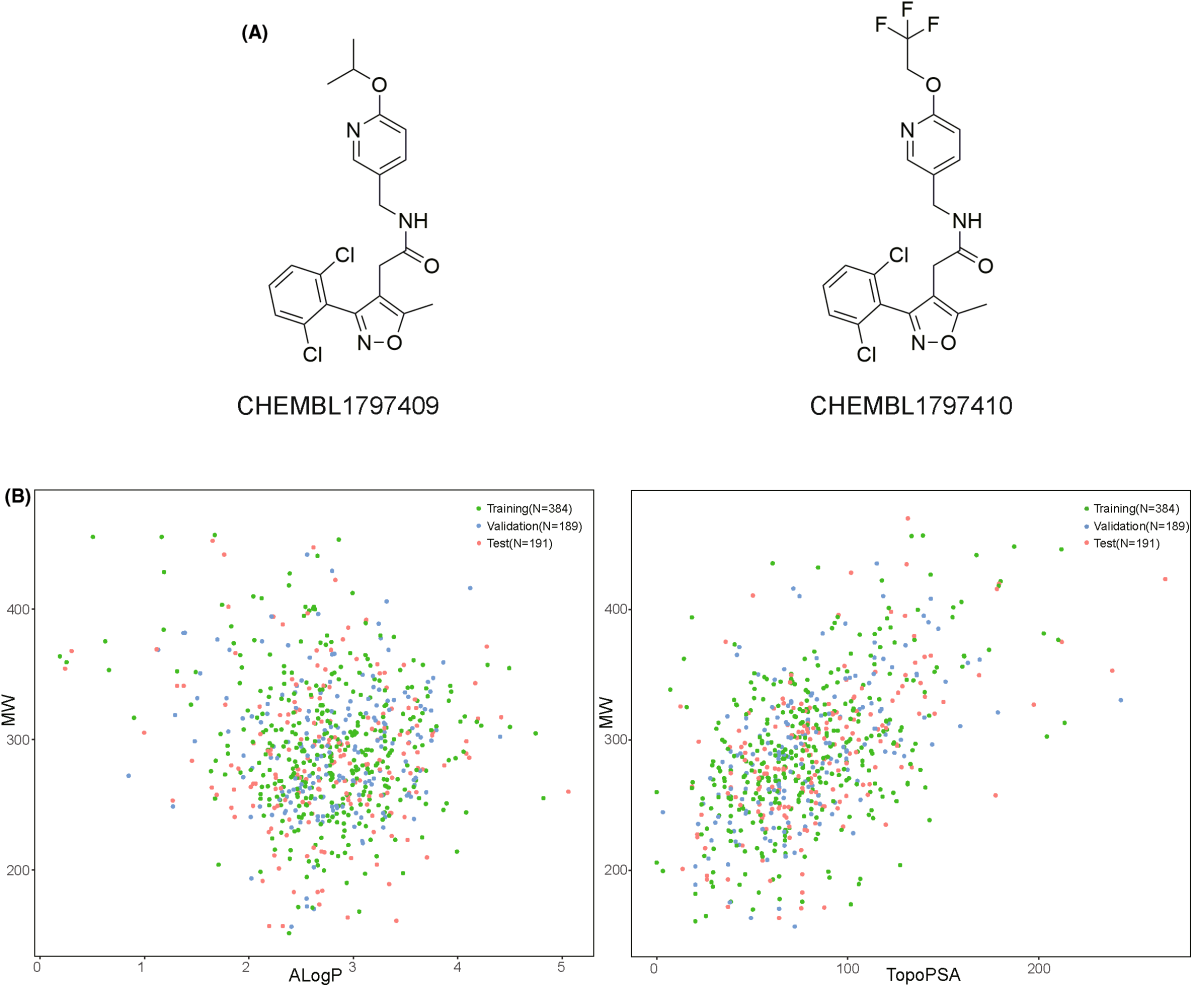
(A) Two similar molecules from the same study. (B) Chemical diversity analysis of training, test and validation datasets. The *y*‐axis is MW, where the *x*‐axis on the left is ALogP and the *x*‐axis on the right is TPSA. Green, blue and red points represent the samples in the training set, validation set and test set, respectively.

In general, Na_V_1.5 inhibitors reported in the same research paper often have very similar structures. If these molecules appear in both the training set and test set, a “data leakage” problem would arise. As shown in Figure 1A, two molecules reported in the same article were highly similar and the Tanimoto similarity (using the ECFP4 fingerprint to obtain the similarity) of them was 0.9590.^19^ Hence, hierarchical clustering was performed on the selected positive molecules according to the inter‐group Tanimoto distance (1‐Tanimoto similarity). In clustering, we used the hierarchical clustering method. Three functions from the RDkit package (http://www.rdkit.org/) in python were employed in this process including BulkTanimotoSimilarity, ForwardSDMolSupplier and GetMorganFingerprintAsBitVect. Then undersampling was performed by setting a certain cutoff value in the clustering tree to reduce molecular similarity among positive molecules. To make sure the number of left positive molecules was not too small to build machine learning models, the cutoff value was set from 0.1 to 0.6, and then a suitable value was chosen.

In the drug screening process, to distinguish effective inhibitors from thousands of negative molecules, the negative molecules would have wider chemical space. We try to consider this point in the model training and evaluation processes. So there were some molecules randomly extracted from the ChEMBL database were also included as another part of negative molecules. The “chembl_webresource_client” package (https://github.com/chembl/chembl_webresource_client) in Python was used to implement this process. Then these randomly extracted molecules were processed similarly as mentioned above^1^: the compounds with atom number over 120 were deleted and^2^ only molecules confirmed by Linpinski's rule of 5 were left.

Following the previous process, a positive dataset containing 364 molecules and a negative dataset containing 400 (96 + 304) molecules were obtained. Then, the similarity of negative molecules were visualization by Tanimoto similarity and heatmap. The positive and negative datasets were then divided into a training set, a validation set and a test set using the proportions of 2:1:1, as shown in Table 1.

**TABLE 1:** Number of molecules in the training set, validation set and test set.

	Positive molecules	Negative molecules	Total
Training set	184	200	384
Validation Set	89	100	189
Test set	91	100	191
Total	364	400	764

### 2.2 Sample distribution and similarity test

The distribution of samples would influence the quality of classification models. To test the distribution of the datasets, three descriptors of these molecules including molecular weight (MW), GhoseCrippen LogKow (ALogP) and Topological polar surface area based on fragment contributions (TPSA) were plotted as scatter plots to show the distribution. The AlogP value represents the partition coefficient between octanol and water, which is crucial for the hydrophobicity of the molecule. It is based on the Ghose–Crippen method,^20^ which is calculated from a regression equation based on the hydrophobicity contributions of 120 atom types, including bonding of H, C, N, O, S and halogens. Further, molecular Tanimoto similarity characterized by ECFP4 fingerprints was employed to test the similarity of the samples. The heat maps describing the overall similarity of 100 molecules randomly selected from the training set were used to visualize molecular similarity. The average similarity was also calculated to evaluate the similarity of these molecules.

### 2.3 Extraction of molecular features

Firstly, six molecular descriptors were generated using PaDEL‐Descriptor to perform a simple comparison of positive and negative molecules. These six descriptors included molecule weight (MW), atomic polarizability (apol), the logarithm of 1‐octanol/water partition coefficient (ALogP), the number of hydrogen bond donors (nHBDon), the number of rotatable bonds (nRot) and the number of hydrogen bond acceptors (nHBAcc),^21^, ^22^, ^23^ five of which were Linpinski's rule of five. A *T*‐test was then used to evaluate the results. After comparison, to construct effective classifiers, six kinds of molecular fingerprints were used, which were generated by PaDEL‐Descriptor software. These six fingerprints included CDK fingerprints (CDK, 1024 bits), Estate fingerprints (Est, 79 bits), Extended fingerprints (Ext, 1024 bits), Graph only fingerprints (GO, 1024 bits), MACCS fingerprints (MACCS, 166 bits) and PubChem fingerprints (Pub, 881 bits).

### 2.4 Establishment of classification models

Five machine learning algorithms implemented by “sklearn” Python package (http://www.scikit‐learn.org/) were used to construct machine learning models, namely logistic regression (LR), support vector machine (SVM), naive Bayes (NB), Multilayer Perceptron (MLP) and random forest (RF).^24^ The 5‐fold cross validation (CV) and grid search were used to find the best parameters of these classifiers in the training set.

Logistic regression (LR) is an algorithm which uses least squares for developing a linear model describing a response from an explanatory variable(s).^25^ In this case, a generalized linear model is established using the Sigmoid function as the connection function.

Support vector machine (SVM) aims to find a hyperplane in the multi‐dimension vector space in which each dimension represents a feature to classify two classes. In multi‐dimensional problems, it uses kernel functions to map data to a feature space in which a linear separator can be found.^26^ In this study, different molecular fingerprints consist of multi‐dimensional features where positive dataset and negative dataset are distributed in different areas divided by an unknown hyperplane.

Naive Bayes (NB) is a simple approach using Bayes' theory to find the best classification method.^27^ Bayes' theory aims to make the optimal decision by generating the posterior class probability of a test data on the basis of class conditional density estimation and class prior probability.^28^

Multilayer perceptron (MLP) is a kind of machine learning method which has a multi‐layered neuron structure. This model is suitable for nonlinear fitting, because of a lot of parameters. Many research proved that MLP methods are suitable for drug‐target interaction prediction.^29^, ^30^ When the number of training data is big, the deep learning models based on multilayer perceptron could always have good prediction results.^31^

Random forest (RF) is an ensemble method consisting of many individual decisions. The final predicted label depends on the vote of each decision tree.^32^ The decision tree method is a commonly used data mining method to build classification models.^33^

Graph convolutional artificial neural networks (ANN) can treat the structure of molecules as a network, transform molecules into structure matrices and feature matrices and perform feature transfer and model training on the molecular structure. Related research shows that the graph convolution method has achieved good results on very large sample volume molecular property prediction tasks, but it is not in general as good as traditional ML methods such as SVM on certain specific data sets.^34^ The GraphConvModel function of the “DeepChem” package (https://www.deepchem.io/) in Python was used to establish a graph convolutional ANN model for comparison with other classification models.^35^ The setting of parameters are batch_size = 10, mode = ‘classification’, nb_epoch = 10.

### 2.5 Model evaluation

The validation set and test set were used to evaluate the performance of these different models. Prediction accuracy (Q), sensitivity (SE), specificity (SP) and Matthew's correlation coefficient (MCC) were employed to evaluate the classification models, as shown below.^36^ Among them, the MCC evaluation index is a common classifier evaluation index calculated based on the confusion matrix. MCC value was used as a determining evaluation index^36^ in our research. TP, TN, FP and FN were parameters which represent the number of true positives, the number of true negatives, the number of false positives and the number of false negatives, respectively. Further, the receiver operating curves (ROC) was plotted which described the relation of FPR (False Positive Rate) and TPR (True Positive Rate) in different models and the PR curves which described the Precision and TPR were also plotted. To evaluate the models, the area under the curve (AUC) of both curves was used.

$$Q=\frac{\mathrm{TP}+\mathrm{TN}}{\mathrm{TP}+\mathrm{TN}+\mathrm{FP}+\mathrm{FN}}$$

$$\mathrm{SE}=\frac{\mathrm{TP}}{\mathrm{TP}+\mathrm{FN}}$$

$$\mathrm{SP}=\frac{\mathrm{TN}}{\mathrm{TN}+\mathrm{FP}}$$

$$\mathrm{MCC}=\frac{\mathrm{TP}\times \mathrm{TN}-\mathrm{FP}\times \mathrm{FN}}{\sqrt{\left(\mathrm{TP}+\mathrm{FP}\right)\left(\mathrm{TP}+\mathrm{FN}\right)\left(\mathrm{TN}+\mathrm{FP}\right)\left(\mathrm{TN}+\mathrm{FN}\right)}}$$

we want to find models which have good prediction performance both in the validation set and test set. Then, the top 10 models in the validation set were selected based on the MCC to do prediction in the test set. In addition, the top 5 models in the validation set were used to build an ensemble learning model to improve the prediction performance.^37^ The prediction result of the ensemble learning model was simple majority voting of the top 5 classification models. In other words, the final classification of a new sample would depend on the number of “1” labels in the five basic classifiers. For example, if there were 3/5 basic learners predicting “1” label for the sample, the final predicted label would be “1.”

### 2.6 Privileged substructure analysis

The privileged substructures (alert substructure) were the potential fragments of chemicals that may bind to target proteins and operate functions. Molecules containing such fragments are of particular interest to researchers. Hence, the “bioalerts” package (https://github.com/isidroc/bioalerts) was used to extract the alert structure of the Na_V_1.5 ion channel. All datasets in this study were utilized to analyse privileged substructures. The fingerprint used in this method was ECFP4. Positive molecules and negative molecules were counted by setting searching radius (radi = 2, 3 and 4). The probability for a substructure to be a structural alert was derived from the probability density function of the binomial distribution in the positive and negative groups. These were used to calculate a P value^38^ which indicated the level of significance when considering a given substructure as a structural alert. Only when the possibility of a certain substructure occurring in positive molecules was significantly larger than that occurring in negative molecules, the substructure would be then recognized as an alert substructure.

## 3 RESULTS

### 3.1 Data set preparation and analysis

As for positive molecules, we obtained 2145 molecules with IC50 values from the ChEMBL database (Figure S1A). Firstly, we deleted duplicate molecules and only keep the molecules which were verified experimentally. In this step, 1957 molecules left (Figure S1A). Then, we kept the molecules with IC50 less than 30,000 nM and 1758 molecules left. In this step, there are 172 molecules with IC50 greater than 30,000 nM which were regarded as part of negative molecules (Figure S1B). In 1758 positive molecules, there are 1558 molecules with N(atom) < 120 and they meet Linpinski's rule of five (Figure S1A). So these 1558 molecules were used to do clustering and undersampling to reduce molecular similarity. After undersampling, only 364 molecules were left.

As for negative molecules, the first part is 172 molecules as mentioned above. In these molecules, 96 molecules meet Linpinski's rule of five and N(atom) < 120. In addition, we also extract molecules from ChEMBL randomly to extend the negative molecule set. We randomly extracted 304 molecules which met Linpinski's rule of five and N(atom) < 120. These 304 molecules were another part of the negative molecules. In total, we got 400 (96 + 304) molecules.

These positive and negative molecules were then divided into a training set, a validation set and a test set in the proportions of 2:1:1 as shown in Table 1. Then MW, ALogP and TPSA were used as the main representation of the chemistry space to show the diversity of samples. As shown in Figure 1B, these three datasets are almost spread uniformly in the same chemistry space which proved the rationality of the sampling process.

### 3.2 Result of molecular similarity test

**FIGURE 2:**
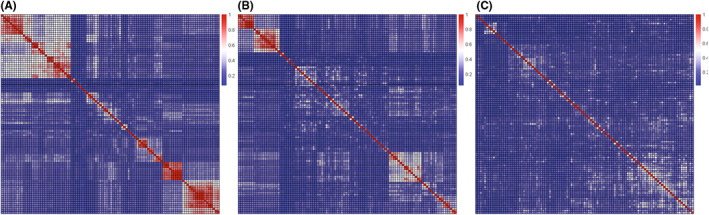
The Tanimoto similarity heatmap of 100 positive and 100 negative molecules. (A) Molecular similarity result of positive molecules before excluding similar molecules. The average similarity of all positive molecules is 0.301. (B) Molecular similarity result after excluding similar molecules. The average similarity of all positive molecules is 0.238. (C) Molecular similarity result of negative molecules. The average similarity of all negative molecules is 0.204.

When evaluating molecular similarity, Tanimoto similarity based on ECFP4 fingerprint was used. We used the hierarchical clustering method to perform undersampling to reduce molecular similarity and set the method parameter as “average”: the between‐group distance (Tanimoto distance = 1 − Tanimoto similarity) is equal to the average distance between the two group objects. The cutoff value in the clustering tree was set to a range from 0.1 to 0.8, as shown in Figure S2. When the cutoff value increases, the number of left positive molecules decreased. And when the cutoff value changed from 0.4 to 0.5, the decreasing speed of positive molecules reached the maximum. To avoid too little molecules left, 0.4 was chosen as the final cutoff value. And after clustering and undersampling, there were 364 positive molecules left (Table 1 and Figure S1). Figure 2 showed the similarity of 100 randomly selected positive or negative molecules. The average similarity value of the whole positive molecule set before undersampling samples is 0.301 (Figure 2A); the average similarity value after undersampling s is 0.238 (Figure 2B) and the average similarity of the whole negative molecule set was only 0.204 (Figure 2C). And compared with other research results,^39^, ^40^, ^41^ the molecule similarity here (0.238 for positive molecules and 0.204 for negative molecules) has lower values. The above results showed that clustering and undersampling can effectively reduce molecular similarity in the positive molecules and there is no need to do clustering and undersampling in the negative molecules. Building models based on data which have wider chemical space can make the models have wider application range.

### 3.3 Distribution analysis of molecular descriptors

**FIGURE 3:**
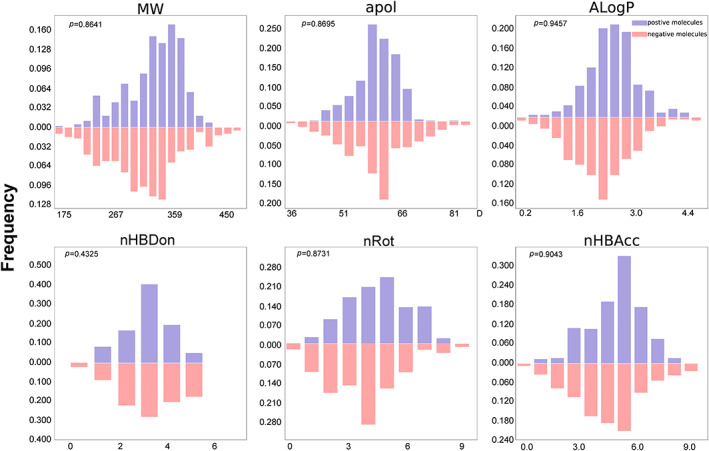
The distribution of positive blockers and negative compounds in six descriptors. The molecular descriptors are MW, apol, ALogP, nHDon, nRot and nHBAcc. Blue bars represent positive molecules and red bars represent negative molecules.

Six molecular descriptors were used to analyse the distribution of 6 descriptors between positive and negative molecules using t‐test. The *p* Values in MW, apol, AlogP, nHBDon, nRot, and nHBAcc are 0.8641, 0.8695, 0.9457, 0.4325, 0.8731 and 0.9043, respectively, which proved that positive and negative molecules have a similar distribution in these descriptors and cannot be distinguished only by some simple features, as shown in Figure 3. Hence, it is necessary to build the proposed classification models based on molecular fingerprints.

### 3.4 Evaluation of classification models

**FIGURE 4:**
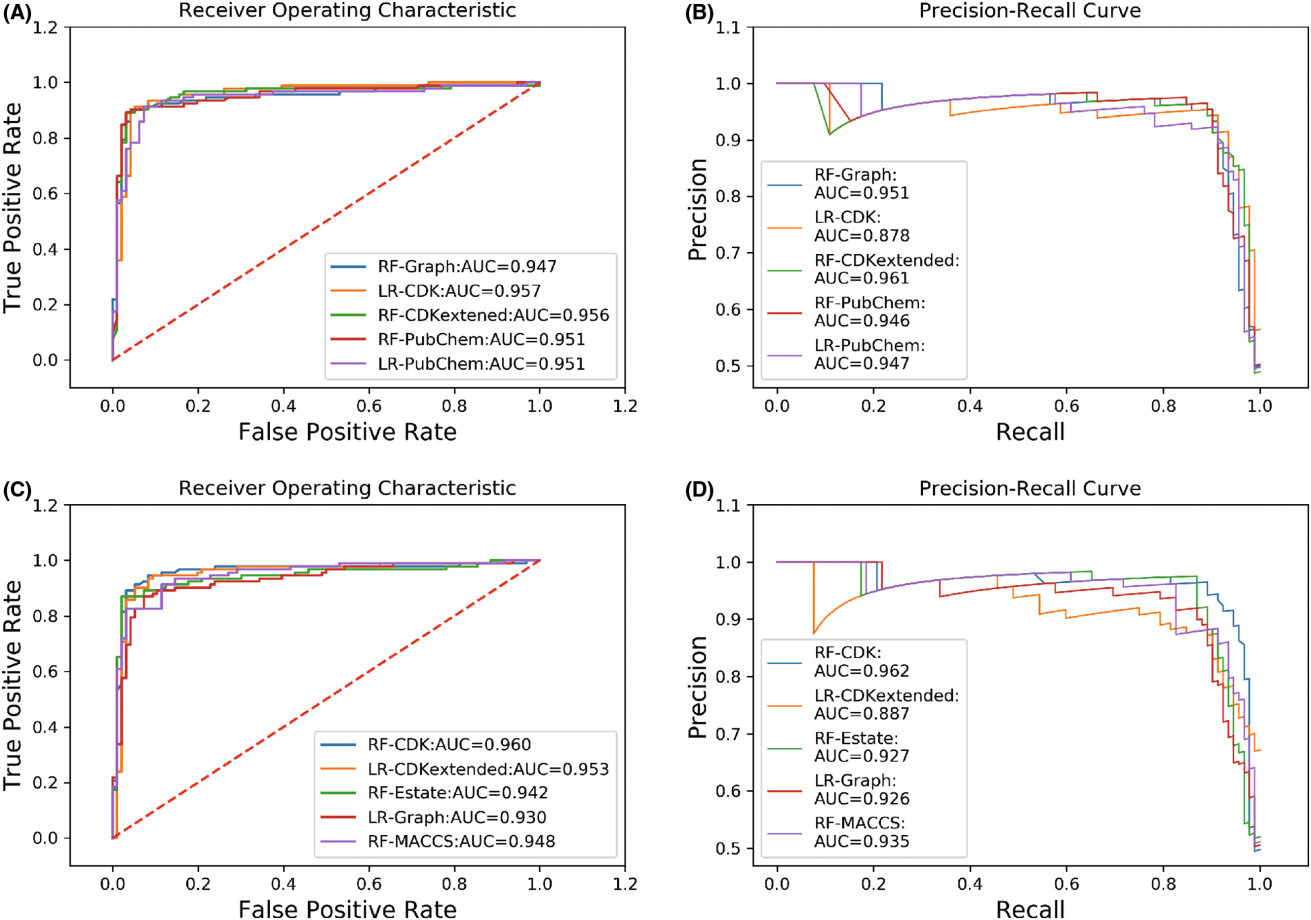
The ROC and PR curve of the top ten models in the test set. (A) The ROC curves of the top five models with biggest MCC and their AUC in the test set. (B) The PR curves of the top five models with biggest MCC and their AUC in the test set. (C) The ROC curves of the top 6–10 models and their AUC in the test set. (D) The PR curves of the top 6–10 models in the top ten models and their AUC in the test set.

The result of cross validation was shown in Table S1. Then, the best model in every machine learning method was used to establish models using the whole training set. The performance of the developed models on the training and validation sets is shown in Table S2 and Table S3 and was ranked by MCC. The best model in the training set is RF_Graph (MCC = 0.887, AUC = 0.966, Table S2), while the MCC values of RF_Graph in the validation set and test set were 0.885 (Table S3) and 0.863 (Table 2), which were not significantly less than 0.887. So in this condition, we did not meet overfitting problem in this task. In the validation set, the top ten models based on MCC value for the validation dataset are RF‐Graph, LR‐CDK, RF‐CDKextended, RF‐PubChem, LR‐PubChem, RF‐CDK, LR‐CDKextended, RF‐Estate and LR‐Graph, RF‐MACCS. Then, the top ten models were chosen and evaluated in the test set (Table 2). The RF‐Graph model had the best result for the test dataset: Q was 0.931, SE was 0.902, SP was 0.958, AUC of the ROC curve was 0.947 and MCC was 0.863. In both the test set and the validation set, the best model was RF‐Graph, which indicated that this model could be an excellent classifier for this study. The MCC values of the DeepChem model on the validation dataset and the test dataset were 0.570 and 0.586, respectively (Table 3). The ensemble model has an MCC value of 0.854 in the validation set and 0.874 in the test set (Table 3). Compared to the results of the RF‐Graph, there is no obvious improvement in the ensemble model. In addition, the ROC curves and PR curves of the top ten models in the test set are shown in Figure 4. It can be seen that the gap between the ROC curves and the PR curves is not obvious for the top 10 models.

**TABLE 2:** Top ten classification models in the test set.

	Q	SE	SP	AUC*	MCC
RF_Graph	0.931	0.902	0.958	0.947	0.863
LR_CDK	0.931	0.913	0.948	0.957	0.862
RF_CDKextended	0.926	0.88	0.969	0.956	0.854
RF_PubChem	0.926	0.88	0.969	0.951	0.854
RF_CDK	0.92	0.87	0.969	0.96	0.844
LR_CDKextended	0.92	0.935	0.906	0.953	0.841
LR_PubChem	0.915	0.902	0.927	0.944	0.83
RF_Estate	0.91	0.891	0.927	0.942	0.819
LR_Graph	0.894	0.891	0.896	0.93	0.787
RF_MACCS	0.862	0.837	0.885	0.948	0.724

**TABLE 3:** Evaluation results of DeepChem model and ensemble models.

id	Q	SE	SP	AUC*	MCC
deepchem_validation	0.784	0.731	0.832	0.854	0.570
deepchem_test	0.782	0.630	0.927	0.837	0.586
ensemble_validation	0.937	0.914	0.959	‐	0.854
ensemble_test	0.928	0.891	0.969	‐	0.874

### 3.5 Analysis of privileged substructures

The fragments with *p* < 0.05 would have a high frequency to bind to the target protein, and they were considered as an alert substructure. The privileged substructures were sorted according to the P value from small to large. As shown in Table S4, these privileged substructures included some typical fragments of sulfonamides and some fragments which have a large structural Steric hindrance. They are more common in Na_V_1.5 blockers, indicating that chemicals that contain them may have a high possibility to inhibit Na_V_1.5. Previous research has partially revealed the reason they can inhibit the Na_V_1.5 effectively. There are two possible mechanisms. One is that some structure with a large Steric hindrance blocks the pore physically where sodium ions pass through.^42^ This theory fits fragments S5, S8, S9 and S6. The other theory is that some chemicals change the conformation of the channel by binding with some peptide residue through van der Waals interactions and salt bridge, which fits some chemicals with sulfa fragments such as S1, S3, S6 and S7.^43^

**FIGURE 5:**
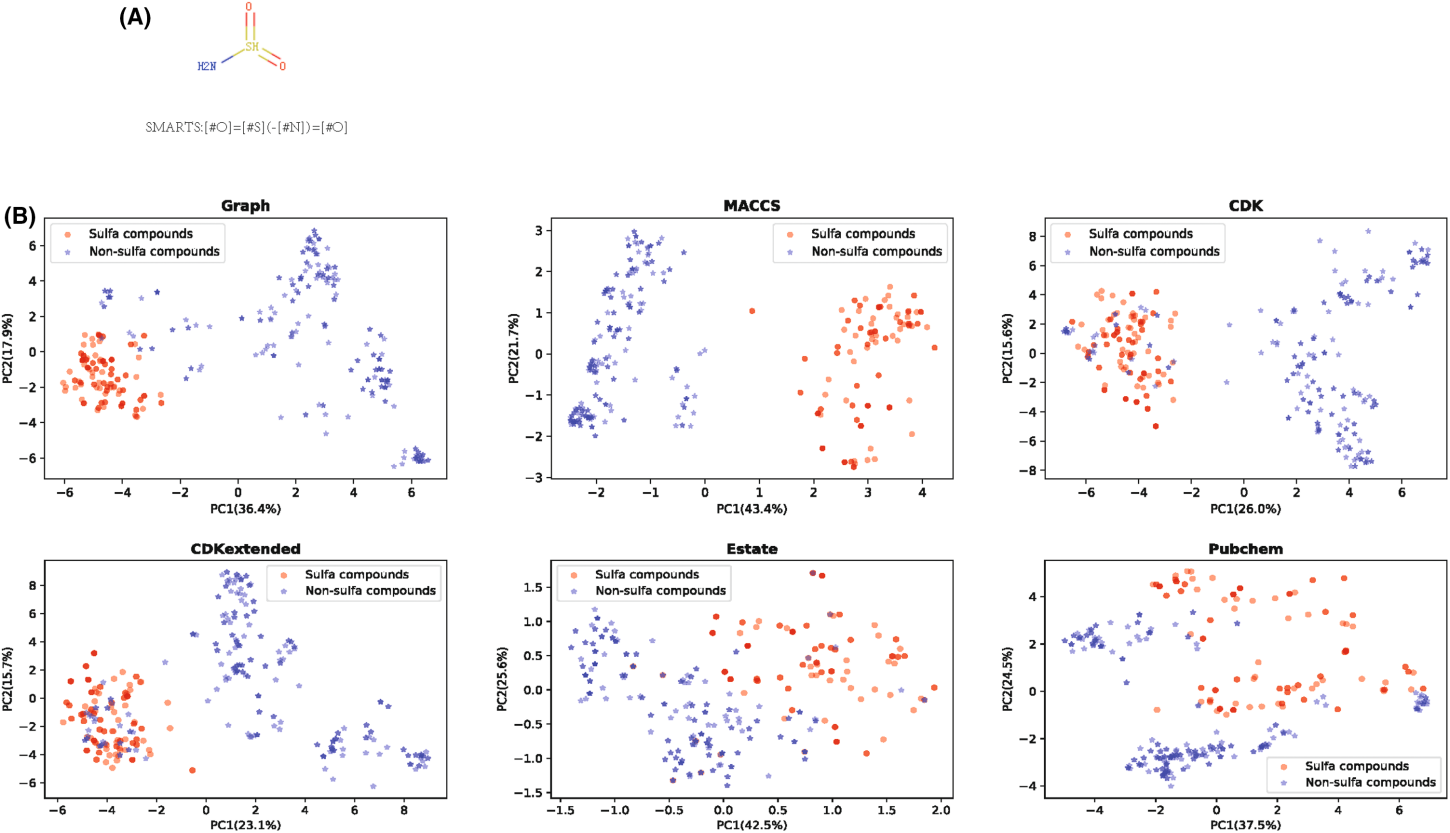
(A) The characteristic structure of sulfa drugs and its SMARTS code. (B) Principal component analysis results of positive molecules. The red dots are sulfa drugs and the blue dots are non‐sulfa drugs. The x‐axis is the first principal component (variance contribution rate) and the y‐axis is the second principal component (variance contribution rate).

We group positive molecules based on the alert substructure. We converted all positive molecules from SMILES to SMARTS to find molecules containing the characteristic structure of sulfa drugs. The characteristic structure of sulfa drugs and their SMARTS are shown in Figure 5A. In the end, we obtained 235 sulfa drugs and 129 non‐sulfa drugs from 364 positive molecules. We used six kinds of fingerprints to do the principal component analysis of positive molecules, and the relevant results are shown in Figure 5B. It can be seen that Graph and MACCS molecular fingerprints can better distinguish sulfonamides and non‐sulfonamides in this unsupervised learning task. Among them, MACCS has obvious classification boundaries. Other molecular fingerprints have found no clear boundaries for these two kinds of drugs.

## 4 DISCUSSION

As a critical molecule in the regulation of cardiac electrophysiology, Na_V_1.5 has been a focal point in related research. As previously discussed, researchers have paid great attention to the potential cardiac risk caused by some Na_V_1.5 blockers to instruct on rational drug use. In addition, some researchers concentrate on the therapeutic effect on cardiac arrhythmias, the balance between therapeutic and adverse effects being the important issue.^44^ Many Na_V_1.5 blockades have been found to have the antiarrhythmic effect, such as lidocaine and phenytoin.^45^, ^46^ These induce the excitability of cardiomyocytes by blocking Na_V_1.5 to relieve arrhythmia. So Na_V_1.5 is a key target in arrhythmia.

At present, there are several methods for screening Na_V_1.5 ion channel drugs, such as the fluorescence resonance energy transfer method, patch‐clamp electrophysiological method and fluorescence imaging plate reader.^47^, ^48^, ^49^ The patch‐clamp electrophysiological method is still the gold standard for ion channel drug screening. However, due to the limitations of experimental equipment and the lack of professionals, screening ion channel drugs using electrophysiological methods often requires significant time and resources. Hence, we need to use cheminformatics methods to accelerate this process. In this study, the RF‐Graph model had the best performance. This model will greatly reduce experimental time and cost. In addition, researchers hope to find specific Na_V_1.5 inhibitors, which often can have huge application potential. To achieve this goal, multiple models can be constructed of different sodium ion channels (Na_V_1.7, Na_V_1.6, etc.). When these models are used to screen compounds at the same time, Na_V_1.5‐specific inhibitors can be obtained.

Compared with other studies, it can be seen that the RF‐Graph model has achieved higher MCC and AUC values, which may be related to the strict data processing and undersampling process.^50^, ^51^ All the top 10 models in the test set are LR and RF based (Table 2). This shows that LR and RF are suitable for the rapid construction of classification models with small sample sizes. In the prediction problem of Na_V_1.5 inhibitors, the RF‐Graph model is better than the graph convolutional ANN model (Table 3), which is consistent with the research results of Korolev et al.^34^ Previous research^52^, ^53^ have shown that GCNNs are not effective at long range information propagation. However, we have not explored this aspect in our current study.

There are still areas for improvement for further work. All the data used originates from the ChEMBL database, with no data set from other sources. After screening the original data, only 364 positive molecules were obtained. The lack of experimental data also greatly limits the applicability of the model. In addition, there is scope for trying different molecular feature extraction methods and ML methods to make the predicted results more reliable. Therefore, combining ML methods to predict Na_V_1.5 inhibitors with experimental high‐throughput screening methods is planned for a future study.

## 5 CONCLUSION

Based on molecular fingerprinting and machine learning methods, 30 classification models were developed and implemented which predict the binding capacity with NaV1.5 protein. In all cases, the most suitable model obtained for the test set was RF‐Graph, of which the Q, SE, SP, AUC and MCC values were 0.9309, 0.9022, 0.9853, 0.9473 and 0.8627. These results are significantly better than the classification model based on graph neural networks. We also extracted 10 kinds of alert substructures which were firmly related to the affinity of inhibition to Na_V_1.5. In the unsupervised learning task of identifying sulfa drugs, MACCS and Graph fingerprints have good results. To conclude, the model established in this research can effectively shorten the development time and cost of Na_V_1.5 inhibitors and provide guidance for related experimental work.

## AUTHOR CONTRIBUTIONS

**Weikaixin Kong:** Conceptualization (equal); data curation (equal); formal analysis (equal); investigation (equal); writing – original draft (equal). **Weiran Huang:** Conceptualization (equal); data curation (equal); formal analysis (equal); methodology (equal). **Chao Peng:** Conceptualization (equal); data curation (equal); formal analysis (equal). **Zhuo Huang:** Conceptualization (equal); funding acquisition (equal); project administration (equal); supervision (equal); writing – original draft (equal); writing – review and editing (equal). **Bowen Zhang:** Methodology (equal); writing – original draft (equal). **Guifang Duan:** Writing – original draft (equal); writing – review and editing (equal). **Weining Ma:** Methodology (equal); writing – review and editing (equal).

## FUNDING INFORMATION

This work was supported by Chinese National Programs for Brain Science and Brain‐like intelligence technology No.2021ZD0202102 to Z.H; National Natural Science Foundation of China Grant Nos. 31871083 and 81371432 to Z.H. and 32000674 to GFD.

## CONFLICT OF INTEREST

The authors declare no competing financial interest.

## Supporting information


Figure S1.

Figure S2.

Table S1.

Table S2.

Table S3.

Table S4.

## Data Availability

All data can be found in public databases.
