# Supplementary figures and images for: Multiple machine learning methods aided virtual screening of NaV1.5 inhibitors
Source: J Cell Mol Med. 2022 Dec 27;27(2):266–76. doi: 10.1111/jcmm.17652 (PMC9843531; doi:10.1111/jcmm.17652)

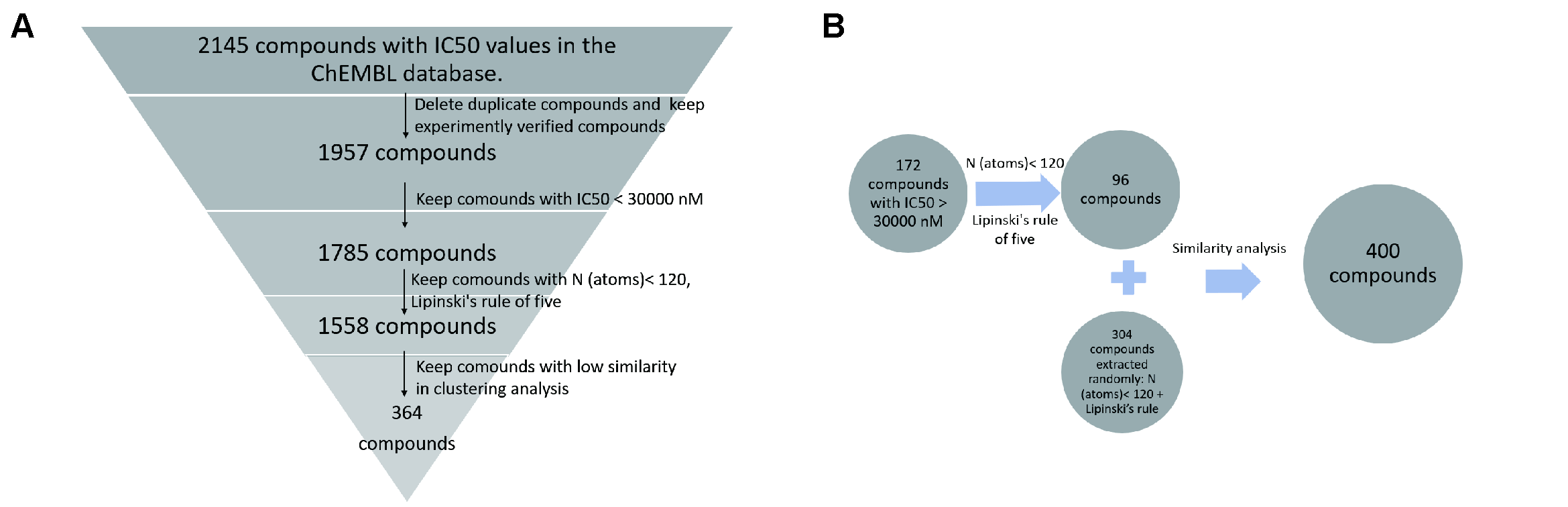

Supplement: Supplementary file 1 — Figure S1. [file JCMM-27-266-s002.tiff]

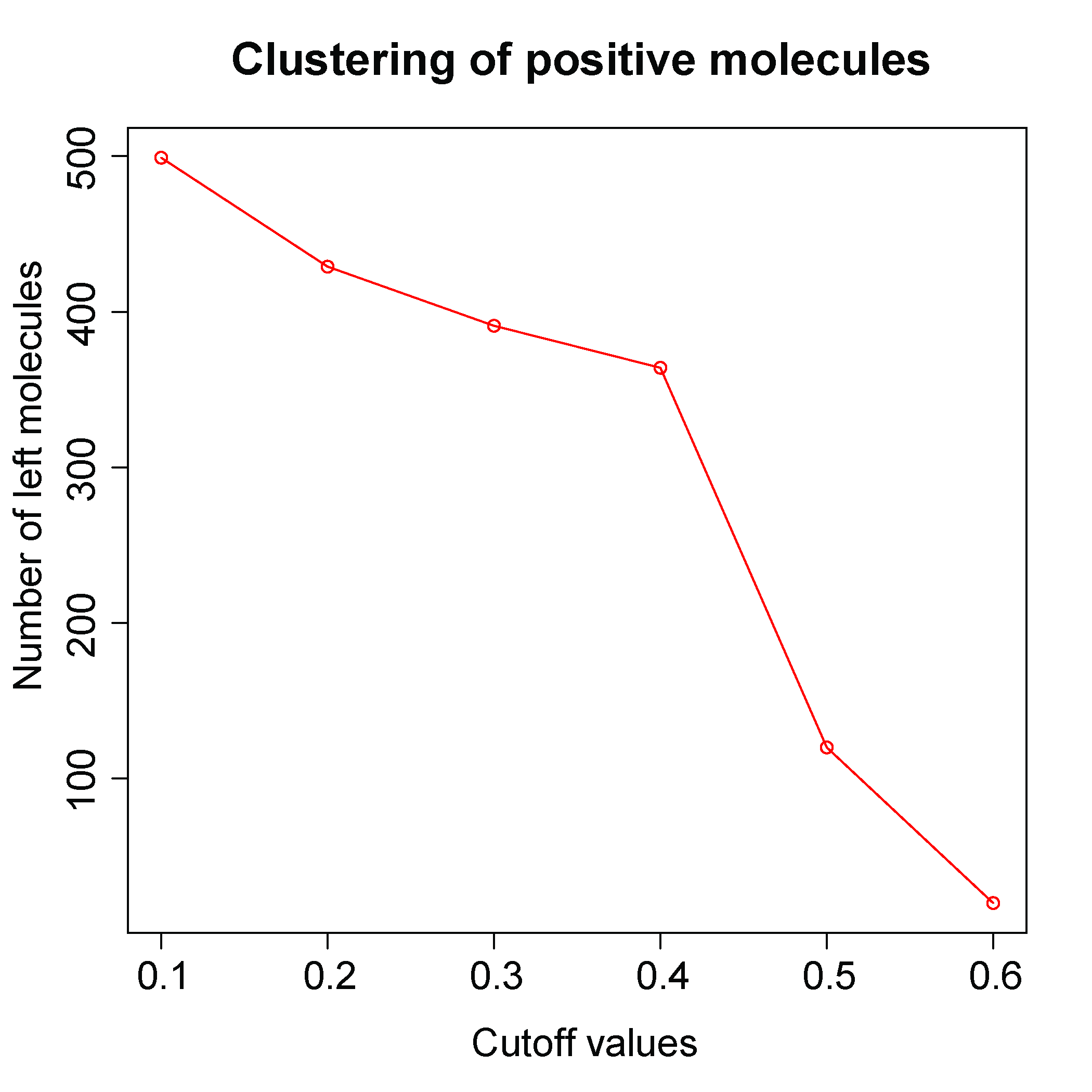

Supplement: Supplementary file 2 — Figure S2. [file JCMM-27-266-s004.tiff]
