# Supplementary material for: Multiple machine learning methods aided virtual screening of NaV1.5 inhibitors
Source: J Cell Mol Med. 2022 Dec 27;27(2):266–76. doi: 10.1111/jcmm.17652 (PMC9843531; doi:10.1111/jcmm.17652)
Supplement: Supplementary file 6 — Table S4. [file JCMM-27-266-s005.pdf]

Supplementary Table 4 The privileged substructures of NaV1.5 obtained by bioalerts

Priviledge Structures	Structure ID	Radi	p_value	Compounds	Positive Compounds	Negative Compounds
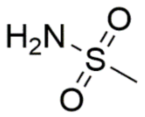	S1	2	<0.05	40	37	3
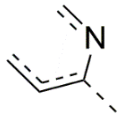	S2	2	<0.05	25	25	0
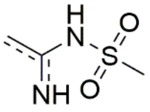	S3	3	<0.05	27	27	0
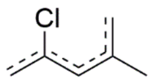	S4	3	<0.05	29	29	0

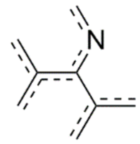	S5	3	<0.05	24	24	0
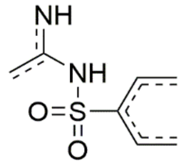	S6	3	<0.05	17	17	0
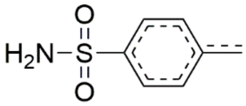	S7	3	<0.05	58	58	0
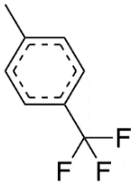	S8	3	<0.05	23	23	0
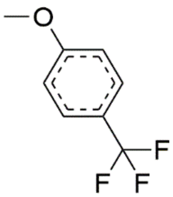	S9	4	<0.05	17	17	0

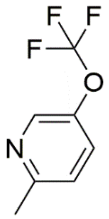	S10	4	<0.05	11	11	0
